# The Promising Role of Probiotics in the Prevention of Cardiovascular Risk Factors and Their Implication in Reducing Coronary Artery Disease: A Literature Review

**DOI:** 10.7759/cureus.86292

**Published:** 2025-06-18

**Authors:** Rahma Hashish, Khaled Agha Tabari, Shivling S Swami, Alousious Kasagga, Amanuel Kefyalew Assefa, Maysaa N Amin, Ann Kashmer Yu

**Affiliations:** 1 Internal Medicine, Sherwood Forest Hospitals NHS Foundation Trust, Sutton-in-Ashfield, GBR; 2 Radiology, Queen Elizabeth University Hospital, Glasgow, GBR; 3 Internal Medicine, California Institute of Behavioral Neurosciences & Psychology, Fairfield, USA; 4 Pathology, Peking University, Beijing, CHN; 5 Orthopaedics and Trauma, University Hospitals of Leicester NHS Foundation Trust, Leicester, GBR; 6 Microbiology/Immunology, California Institute of Behavioral Neurosciences & Psychology, Fairfield, USA

**Keywords:** cardiovascular disease, coronary artery disease, gut microbiota, hypercholesterolemia, hypertension, obesity, pathophysiology, probiotics, type 2 diabetes

## Abstract

Coronary artery disease (CAD) remains one of the most common causes of mortality across the globe, which is strongly associated with modifiable risk factors such as hypertension, hyperlipidemia, type 2 diabetes, and obesity. The role of the gut microbiota in influencing these factors has been established recently. Probiotics, which can modulate gut microbiota, have been investigated as a potential strategy to reduce cardiovascular risk.

This review aims to evaluate current evidence on the role of probiotics in reducing CAD risk factors and to explore the mechanisms through which probiotics may support cardiovascular health.

This narrative review was conducted using studies published within the last five years. The search included databases such as PubMed, Google Scholar, Medline, and ResearchGate. The selection focused on randomized controlled trials (RCTs), meta-analyses, and reviews that examined the impact of probiotics on CAD and its associated risk factors.

Findings from several RCTs and meta-analyses show that probiotic supplementation is associated with improved lipid profiles (including lower LDL-C and triglycerides), better glycemic control, reduced inflammatory markers, and modest reductions in blood pressure and obesity measures. However, results across studies vary due to differences in sample size, duration, probiotic strains, and measured outcomes.

Probiotics may offer a beneficial, non-pharmacological option to support conventional CAD therapies, particularly by targeting key modifiable risk factors. While early results are encouraging, further large-scale, long-term studies are necessary to confirm their clinical effectiveness and guide standardized recommendations.

## Introduction and background

Coronary artery disease (CAD) is a prevalent heart condition in which we can observe the narrowing or blockage of major blood vessels-coronary arteries, caused primarily by plaque-forming atheroma within the intima of the vessel wall [1].

CAD stands as the primary cause of death and a significant factor contributing to the loss of disability adjusted life years (DALYs) globally. A substantial portion of this health burden is concentrated in low- and middle-income countries, which collectively experience nearly seven million fatalities and approximately 129 million lost DALYs each year, as probiotic supplements may have a role in preventing or reducing atherosclerosis and may beneficially affect CAD/CHD (coronary heart disease) [2,3].

There are several risk factors for the development of CAD, which are hyperlipidemia, hyperglycemia, hypertension, inflammation, and oxidative stress, which increase patients' risk of atherosclerosis and, in turn, lead to CAD [4].

In recent years, growing evidence has established the beneficial effect of the gut microbiota in influencing these cardiovascular risk factors. This microbial ecosystem plays a significant role in metabolic regulation, immune function, and systemic inflammation, all of which are significant to the development and progression of CAD. Probiotics, which can modulate the gut microbiota, have therefore been examined as a possible strategy to reduce cardiovascular risk by targeting the underlying metabolic and inflammatory pathways.

The term "probiotic" was first introduced in 1965 by Lilly and Stillwell. Who described them as factors secreted by one microorganism that stimulate the growth of another? The modern era of probiotics is characterized by stating that there are live active cultures used in food fermentation, characterized microbial strains, which are used as additives to food products, and probiotics have also been used in the form of dietary supplements [5].

The gut microbiota is an ecosystem comprising various microbial populations, including bacteria, phages, fungi, yeasts, and viruses [6]. It primarily comprises four main phyla: Firmicutes, Bacteroidetes, Actinobacteria, and Proteobacteria. Notably, Firmicutes and Bacteroidetes account for 90% of the bacterial flora in the colon [6].

One of the mechanisms by which the gut microbiota influences host metabolism is through the production of key microbial metabolites; the short-chain fatty acids (SCFAs) are mainly acetate and propionate. Elevated acetate production from high-calorie diets can lead to increased levels of hormones like ghrelin and insulin, promoting hyperphagia and storage of fat, leading to obesity, hyperlipidemia, and insulin resistance. Acetate promotes cholesterol synthesis; on the other hand, propionate decreases vascular inflammation and inhibits lipogenesis [7].

A study was conducted to explore the different gut microbiota species which are linked to atherosclerosis by testing the fecal samples of adults with coronary atherosclerosis and healthy controls by measuring 16S rDNA, which revealed the presence of Megamonas, Streptococcus, and Veillonella in a significant quantity compared to the healthy group, which is linked to coronary atherosclerosis [7].

The same study also revealed that the abundance of Bifidobacterium, Ruminococcus, and Candidatus Bacilloplasma was significantly lower in adults with coronary atherosclerosis. Ruminococcus decreases the cholesterol in the serum by converting it to bile acids. Bifidobacteria are an important intestinal probiotic present in the intestinal mucus layer, which plays a significant role in preventing coronary atherosclerosis [7].

**Figure 1:**
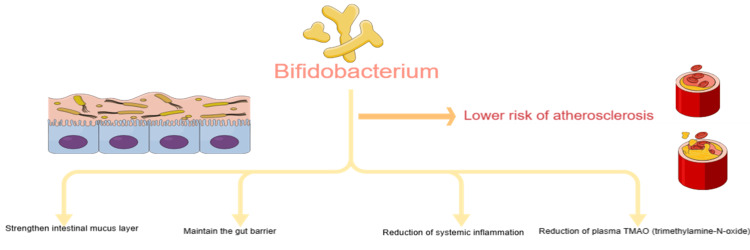
The role of Bifidobacterium probiotics in lowering the risk of coronary atherosclerosis The figure is created by using https://mindthegraph.com

Figure 1 illustrates the mechanism of Bifidobacterium in decreasing the risk of atherosclerosis.

Bifidobacteria, Lactobacilli, and Bacteroides are some of the most well-studied probiotics in experimental and clinical settings. Lactobacillus acidophilus may have a more significant effect on lowering cholesterol than other probiotics. Bifidobacteria have potential benefits in atherosclerosis, especially when combined with lipid-lowering treatment, and probiotics Bacteroides vulgatus and Bacteroides dorei inhibited atherosclerotic plaque formation [8].

The dysbiosis, which is caused by the imbalance between the beneficial and harmful bacteria, is closely linked to cardiovascular health, as highlighted by the gut-heart theory [9]. This theory explores the complex, bidirectional relationship between the gut microbiome, which can influence heart function through producing various metabolites such as trimethylamine N-oxide (TMAO), which is linked to endothelial dysfunction and contributes to atherogenesis, as each 10 μmol/L increase in plasma TMAO levels is associated with a 7.6% increased risk of major cardiovascular events, including CAD [10].

Moreover, the dysbiosis or the leaky gut allows endotoxins such as lipopolysaccharides (LPS) to escape into the bloodstream, which bind to Toll-like receptor 4 (TLR4) on immune cells, accelerating pro-inflammatory cascades that initiate the process of systemic inflammation and endothelial dysfunction, which contribute to the development of atherosclerosis as well [10].

Probiotics prevent the development of CAD by reducing atherosclerosis through modulation of the gut microbiota [11]. Several probiotic formulations have shown promising anti-obesity, anti-inflammatory, and antioxidative effects [12]. This effect of probiotics was demonstrated in recent studies, which showed the glycemic control among type 2 diabetes mellitus (T2DM) patients and improvement of biomarkers of inflammation in patients with CAD, along with lower obesity markers in obese subjects. Moreover, recent studies have proved that probiotics protect against atherosclerosis development [13].

Probiotics are based on the concept that beneficial bacteria are significant for maintaining and restoring gut flora. These bacteria modulate gastrointestinal health by tolerating the acidic and the alkaline media of the gut, sticking to the intestinal epithelium, and inhibiting the harmful bacteria by producing antimicrobial substances [14].

**Figure 2:**
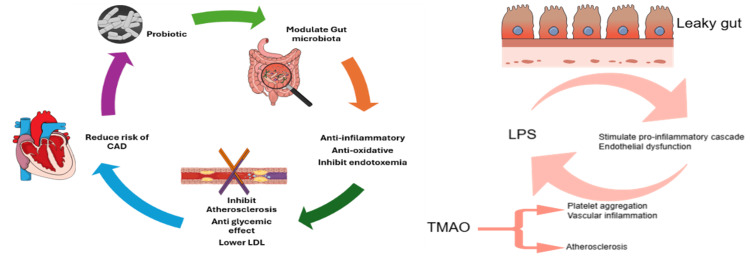
Probiotic-gut-heart axis: Probiotics affect the heart by modulating the gut microbiota through their anti-inflammatory and anti-oxidative properties, which inhibit the plaque formation of atheroma. The role of trimethylamine N-oxide (TMAO) and lipopolysaccharides (LPS) in the development of atherosclerosis. The figure is created by using https://mindthegraph.com and the design of the figure incorporates features from Microsoft Windows SmartArt.

Probiotics can have a protective role against CAD, as selected strains have been shown to reduce inflammation and oxidative stress and enhance lipid profiles and anti-glycemic effect and recent studies showed the effect of probiotics on modulating gut health via reducing chronic inflammation, which reduces endotoxemia, which in turn decreases atheroma formation. Another meta-analysis study showed the probiotics' effect on reducing total cholesterol and low-density lipoprotein cholesterol (LDL-C) quantity among hypercholesterolemic adults who consumed probiotics [15]. Figure 2 illustrates the gut-heart axis.

This review provides clinicians with a consolidated and evidence-based understanding of how probiotics may serve as a supportive intervention in reducing cardiovascular risk factors associated with CAD. By summarizing recent randomized controlled trials (RCTs) and meta-analyses, the paper highlights the potential of probiotics to improve lipid profiles, glycemic control, blood pressure, and systemic inflammation, all of which are critical modifiable contributors to CAD. This review emphasizes recent clinical trials that suggest that probiotics may play a supportive role alongside conventional treatments for CAD, particularly in individuals at elevated risk or those interested in non-pharmacological prevention approaches. In addition to summarizing current evidence, the review draws attention to areas where further research is needed, which may help shape future clinical studies and support the development of more personalized, microbiota-targeted approaches in cardiovascular prevention.

## Review

Methods

This traditional review was conducted following the SANRA guidelines; the selected studies aimed to determine the effect of probiotics on preventing coronary artery disease.

The inclusion criteria for this review comprised English-language, recent full-text articles published within the last five years, including randomized controlled trials conducted on human participants, systematic reviews, meta-analyses, and other relevant types of studies such as literature reviews, mini-reviews, and cross-sectional analyses. In contrast, studies classified as case studies or case reports were excluded from the data.

The study synthesises secondary data from PubMed, Google Scholar, Research Gate, Medline, and Multidisciplinary Digital Publishing Institute. The last date of the search for all databases was March 2025. The search criteria were based on patients with CAD, its associated risk factors, and the effect of probiotics on their prevention and management.

The primary keywords relevant to this research include probiotics, synbiotics, prebiotics, gut microbiota, coronary artery disease, pathophysiology, cardiovascular disease, hypertension, type 2 diabetes, Obesity, and hypercholesterolemia. Boolean operators such as “AND” and “OR” were used to combine keywords and refine results where appropriate.

The studies were mainly gathered from RCTs published in medical journals, which emphasize the protective effects of probiotics on CAD by altering the gut microbiota. This review emphasises the important role of probiotics in shaping the gut microbiota, which promotes cardiovascular health through different pathways and influences the most relevant modifiable risk factors contributing to the development of CAD.

Results

This narrative review highlights the probiotics' protective mechanism and their significant role in preventing CAD and lowering CAD risk factors, as evidenced by including various RCTs and meta-analysis studies conducted in countries with variable populations with CAD or one or more risk factors. It emphasises probiotics’ protective role and mechanism in protecting the cardiovascular system.

In summary, the resulting evidence highlights the significance of probiotics in improving cardiovascular health, which in turn lowers the risk of CAD. However, as few studies have shown, there is no statistical significance on specific parameters when comparing the probiotic and placebo groups. This suggests that further research and broader trials are needed to establish probiotics' protective effect on cardiovascular health. Table 1 summarizes different studies that were included in the review.

**Table 1:** Summary of different trials and studies included in the review. Randomized controlled trials (RCTs) and meta-analysis studies were performed on different participants with coronary artery disease (CAD) or its risk factors such as type 2 diabetes mellitus (T2DM), hypertension (HTN), obesity & hypercholesterolemia, through the effect on different cardiac parameters such as glycated hemoglobin (HBA1C), fasting blood glucose (FBG), high-density lipoprotein (HDL-C), low-density lipoprotein (LPL-C), interleukin 1-beta (IL-B), lipopolysaccharide (LPS) and their reflection on lowering the atherosclerotic cardiovascular disease (ASCVD) and Seattle Angina Questionnaire (SAQ) scores. The probiotic capsule (LactoLevureR) contains Lactobacillus acidophilus (1.75 × 109 colony-forming units (CFU)), Lactobacillus plantarum (0.5 × 109 CFU), Bifidobacterium lactis (1.75 × 109 CFU), and Saccharomyces boulardii (1.5 × 109 CFU).

Author/ Published Year	Type of Trial	Duration of the Trial	Intervention	Population	Age of the Subjects /Years	Country	Outcome
Liu et al. 2024 [12]	RCT	8 weeks	Probiotics +Inulin	116 patients with CAD	35-55	Iran	Significant improvement of the gut microbiota. Decrease inflammation & oxidative stress
Nuankham et al.2024 [14]	RCT double-blind, placebo-controlled	90 days	L. paracasei TISTR 2593 coated in maltodextrin with each capsule containing 1.05 × 10^9^ CFU/g.	22 patients with hypercholesterolemia	30–65 years	Thailand	Lower LDL-C levels. Improvement of hypercholesterolemia
Sun et al. 2022 [16]	RCT	6 months	Probio-M8 as an adjunctive treatment with stains. 20 mg of atorvastatin +two grams per sachet; 3 × 10^10^ CFU/sachet/day.	80 patients with CAD	No age-related criteria	China	Lower IL-6, lower LDL-C. Overall, improvement of CAD-associated symptoms (improved SAQ score) and the protective effect on CAD.
Zikou et al. 2023 [17]	RCT, Double-blinded	6 months	The probiotic capsule (LactoLevure^R^)	91 patients with T2DM	Above 18	Greece	Significant reduction of HBA1C and FBG. Decrease in waist circumference. Overall reduction of diabetes-related complications.
Palathinkara et al. 2025 [18]	Meta- analysis trial	Data from 1999–2020.	Probiotic supplementation OR Food	14,992 survey responders with CVD	Above 18	USA	High HDL-C. Lower HA1C. Lower Triglyceride. Lower ASCVD risk scores.
Moludi et al. 2021 [19]	RCT	12 Weeks	LGG capsule 1.6 × 10^9^ colony-forming unit (CFU)	44 patients with CAD	No age-related criteria	Iran	Decreased IL1-Beta. Decrease LPS levels. Overall, significant improvement of cardiovascular-related factors.
Shirvani-Rad et al. 2021 [20]	Meta-analysis study	2-26 weeks	Probiotic products (dietary or capsules) =10^7^–10^11^ CFU/day)	16676 overweight/obese adults (BMI >25)	Above 18 years.	Different databases	improved overweight/obese indices parameters
Sato et al. 2024 [21]	RCT	16 weeks	Probiotic capsules with 1 × 10^10^ colony-forming units (CFUs) of B. longum BB536 and 5 × 10^9^ CFUs of B. breve MCC1274 (B-3) per 2 capsules. Placebo capsules without bifidobacteria.	100 participants with a BMI of > 23 and < 30.	20 and 64 years of age	Japan (Shinagawa Season Terrace Health Care Clinic)	Decreases visceral fat and total fat area. Lowers triglyceride levels. Overall reduction in BMI and obesity risk.
Peng et al. 2024 [22]	RCT	16 Weeks	Probiotics containing 10^8 CFU/mL of Lactobacillus, 4 times a day.	213 patients with T2DM.	18 years or above.	China	Reductions in HbA1c and FBG in both groups, but there was no significant difference.
Zarezadeh et al. 2023 [23]	A meta-analysis of RCTs	Less than or equal 10 weeks	Dosage of ≥10^10^ colony-forming units (CFU).	14 meta-analyses with 15,494 participants with hypertension	Above 18 years.	China, Iran, USA, Australia, Hungary, Canda, Brazil	Significant reduction in systolic and diastolic blood pressure.

Discussion

Most of the studies evidenced that CAD is strongly associated with altered gut microbiota as the imbalanced shift of the gut microbiota allows the overgrowth of the pathogenic bacteria, causing an inflammatory process and affection of the physiologic status of the host cells, including the endothelial cells through deficiency or excess of specific metabolites, e.g., TMAO, LPS, or indoxyl sulfate, may yield direct toxic effects on the endothelium or indirect toxicity through their modulatory effects on hormones and biologically active compounds of the host organism [24].

Recent findings from bidirectional Mendelian randomization analysis underscore the important role of specific gut microbiota in influencing the risk of CAD. Genera such as Coprococcus, Intestinibacter, Marvinbryantia, and Parasutterella, and Ruminiclostridium are identified as protective factors, whereas Eisenbergiella, Odoribacter, and Oxalobacter are associated with an increased risk [25].

Furthermore, the weighted median method highlights Holdemanella and an unidentified genus (id.2755, OR: 1.119) as potential risk factors. These findings suggest that targeting gut microbiota may provide innovative strategies for preventing and treating CAD [25].

Reports revealed evidence of the probiotic lactobacilli modulating gut microbiota composition. Lactobacillus species, particularly L. casei, L. plantarum, and L. rhamnosus, have proven effective in managing cardiovascular diseases through cholesterol-lowering mechanisms that influence bile acids (BAs) and TMAO. They also play a significant role in obesity regulation by impacting lipid and glucose metabolism, associated signaling pathways, including nuclear factor-kappa B (NF-κB) and peroxisome proliferator-activated receptor alpha (PPAR-α), and metabolites, including BAs and LPS. Additionally, L. fermentum and L. reuteri are important for addressing T2DM by enhancing insulin sensitivity, pathways, including G-protein coupled receptor 43 (GPR43) and phosphatidylinositol 3-kinase protein Kinase B (PI3K/Akt), and metabolites, including LPS, BAs, and SCFAs. Strains like L. brevis and L. helveticus contribute to hypertension management by regulating nitric oxide and angiotensin-converting enzyme activity. These findings underscore the therapeutic potential of specific lactobacilli in promoting metabolic and cardiovascular health [26]. Table 2 compares the effects of different Lactobacillus probiotic strains on cardiovascular health.

**Table 2:** Comparison of the effect of different Lactobacillus strains and their effect through specific signaling pathways. Signaling pathways such as nuclear factor-kappa B (NF-κB), peroxisome proliferator-activated receptor alpha (PPARα), G-protein-coupled receptor (GPCR), phosphatidylinositol 3-kinase, protein kinase B(PI3K/AKT), and inflammatory modulation pathway through tumor necrosis factor alpha (TNF-α) regulate different metabolites, e.g., bile acids (BAs), trimethylamine N-oxide (TMAO), lipopolysaccharide (LPS), short-chain fatty acids (SCFAs), nitric oxide (NO)and angiotensin-converting enzyme (ACE) further lowers the risk of atherosclerosis, obesity, type 2 diabetes mellitus (T2DM) and hypertension which positively affect the heart and lower the CAD risk.

Lactobacillus Species	Pathway	Mechanism	Metabolites	Effect on the Cardiovascular Health
L. Casei, L. plantarum, L. Rhamnosus	NF-κB PPAR-α	Positive effect on Lipid and glucose metabolism	Increase BA synthesis and decrease TMAO &LPS	Lower cholesterol risk & obesity risk
L. Fermentum, L. Reuteri	GPR43 PI3K/Akt	Increase insulin sensitivity	Decrease LPS and increase SCFAs and BA synthesis	Lower T2DM risk
L. Brevis, L. helveticus	Inflammatory pathway, e.g. TNF-α	Inhibit Inflammatory marker expression	Decrease NO & ACE	Lower hypertension risk

**Figure 3:**
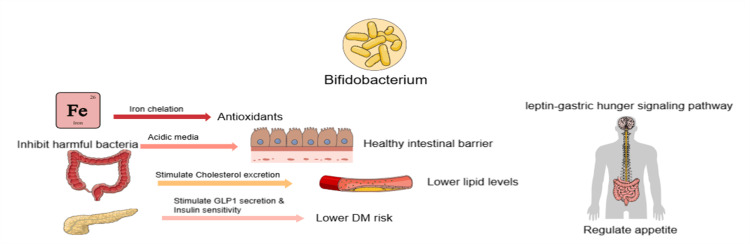
The mechanism of Bifidobacterium probiotics that affect cardiovascular health by lowering diabetes mellitus (DM) risk through the secretion of glucagon-like peptide-1 (GLP-1) and lowering lipid levels The figure is created by using https://mindthegraph.com.

Another probiotic strain found to be important to gut health is Bifidobacterium. It chelates iron, activates antioxidants, and creates an acidic environment to inhibit harmful bacteria. As previously mentioned, it also protects the intestinal barrier, regulates appetite via the leptin-gastric hunger signal communication pathway, and its role in reducing lipid levels by increasing cholesterol excretion. Additionally, it enhances glucose metabolism by stimulating glucagon-like peptide-1 (GLP-1) secretion and insulin sensitivity, lowering diabetes risk [27]. Figure 3 summarizes further the effect of the Bifidobacterium on the cardiovascular system.

Previous studies have provided valuable information about Bacteroides. They showed that Bacteroides were less abundant in obese individuals, had a protective effect against atherosclerosis, and lowered serum LPS activity. Bacteroides could convert cholesterol to coprostanol. The human intestine does not readily absorb Prostanol, so this conversion is considered a natural way to lower serum cholesterol levels in humans [28]. Table 3 summarizes the roles and effects of the most well-studied probiotic strains on gut health and their protective effect on the cardiovascular system.

**Table 3:** Mechanism of the main well-studied probiotic strains and their impact on cardiovascular health. SCFAs: Short-chain fatty acids; TMAO: Trimethylamine-N-oxide; LPS: Lipopolysaccharide; NO: Nitric oxide; ACE: Angiotensin-converting enzyme; IR: Insulin resistance; T2DM: Type 2 diabetes mellitus.

Probiotic Strains	Mechanism	Effect on cardiovascular health
Lactobacilli	Increase production of SCFAs, inhibit the production of TMAO, regulate glucose metabolism and increase insulin sensitivity, and regulate NO & ACE activity.	Lower cholesterol levels, lower T2DM and obesity risk, and lower risk of hypertension
Bifidobacteria	Control the production of TMAO/SCFA, regulate Leptin, reduce glucose metabolism/regulate islet beta cell growth/improve IR production of butyrate iron ion chelator, and promote cholesterol excretion	Regulate gut health, lower obesity risk, lower T2DM risk, anti-inflammatory and anti-oxidative effects, and lower hyperlipidemia
Bacteroides	Lower LPS activity and convert cholesterol to prostanol	Lower serum cholesterol

An RCT showed findings supporting the administration of exogenous probiotics as a promising way to improve the therapeutic effect of CAD. The study concluded that the beneficial effect of the Probio-M8 Co-administration along with the standard routine treatment in patients with cardiovascular disease compared to the standard therapy alone, which was achieved by modulating the gut-heart axis through increasing the production of anti-inflammatory gut organisms and decreasing the levels of TMAO, which relatively decreases the incidence of the cardiovascular events in CAD patients. The same study found that Probio-M8 decreases the serum levels of specific amino acids that are associated with cardiovascular risk, such as l-leucine, l-valine, l-cysteine, and l-arginine [16].

Another mechanism of probiotics is demonstrated through growing evidence that supports the role of the endocannabinoid system (eCB) in the intestinal mucosa, which may be related to dysbiosis-associated low-grade inflammation. Other evidence has demonstrated the association of altered intestinal eCB receptor expression with CAD via CB1 and CB2 receptors. The cardiovascular system cells contain the expression sites of both CB1 and CB2 receptors [12]. Prior studies indicate that probiotic Lactobacillus acidophilus may modulate CB1 receptor expression and enhance tight junction proteins such as occludin and zonulin in a CB1-dependent manner, thereby reducing bacterial translocation. Additionally, CB2 activation has been linked to improved glucose tolerance and gut barrier function in preclinical models [12].

A recent clinical trial indicates that an eight-week coadministration of prebiotic inulin with the probiotic L. rhamnosus (synbiotics) is a more effective strategy than using either alone. This approach significantly reduces chronic inflammation, oxidative stress, and microbial translocation, improving gut microbiota in CAD patients. The endocannabinoid (eCB) system also regulates the function of the gut barrier. The trial shows that prebiotics and probiotics can influence the intestinal eCB system and improve gut permeability. Combining prebiotics and probiotics may enhance traditional cardiovascular treatments, potentially preventing CAD development and progression [12].

A randomized, double-blind, controlled trial conducted in Greece investigated the effects of a six-month regimen of the multi-strain probiotic LactoLevure, which comprises Lactobacillus acidophilus, Lactobacillus plantarum, Bifidobacterium lactis, and Saccharomyces boulardii, on individuals diagnosed with T2DM. The results showed that the probiotic was well-tolerated and improved glycaemic control, lipid profiles, and adiposity measures [17].

A meta-analysis trial was conducted to test the effect of probiotic supplementation on the cardiovascular risk profile in patients with CAD. It found that exposure to probiotic supplementation or probiotic foods was associated with a lower HbA1C percentage, lower circulating triglycerides, and higher HDL-C levels, with an overall lower Atherosclerotic Cardiovascular Disease (ASCVD) risk score [18].

High-fiber foods (e.g., fruits and vegetables) and yogurt are natural sources of prebiotics and probiotics. The use of non-food prebiotics, probiotics, and synbiotics has tripled in the last twenty years due to growing research linking their usage to positive changes in the gut microbiota and various clinical outcomes [29].

**Figure 4:**
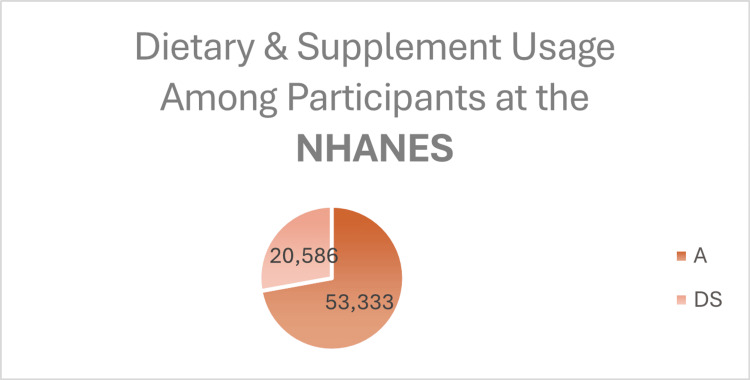
Number of participants who reported using dietary supplements (DS), specifically probiotic supplements, based on the National Health and Nutrition Examination Survey (1999–2018).

**Figure 5:**
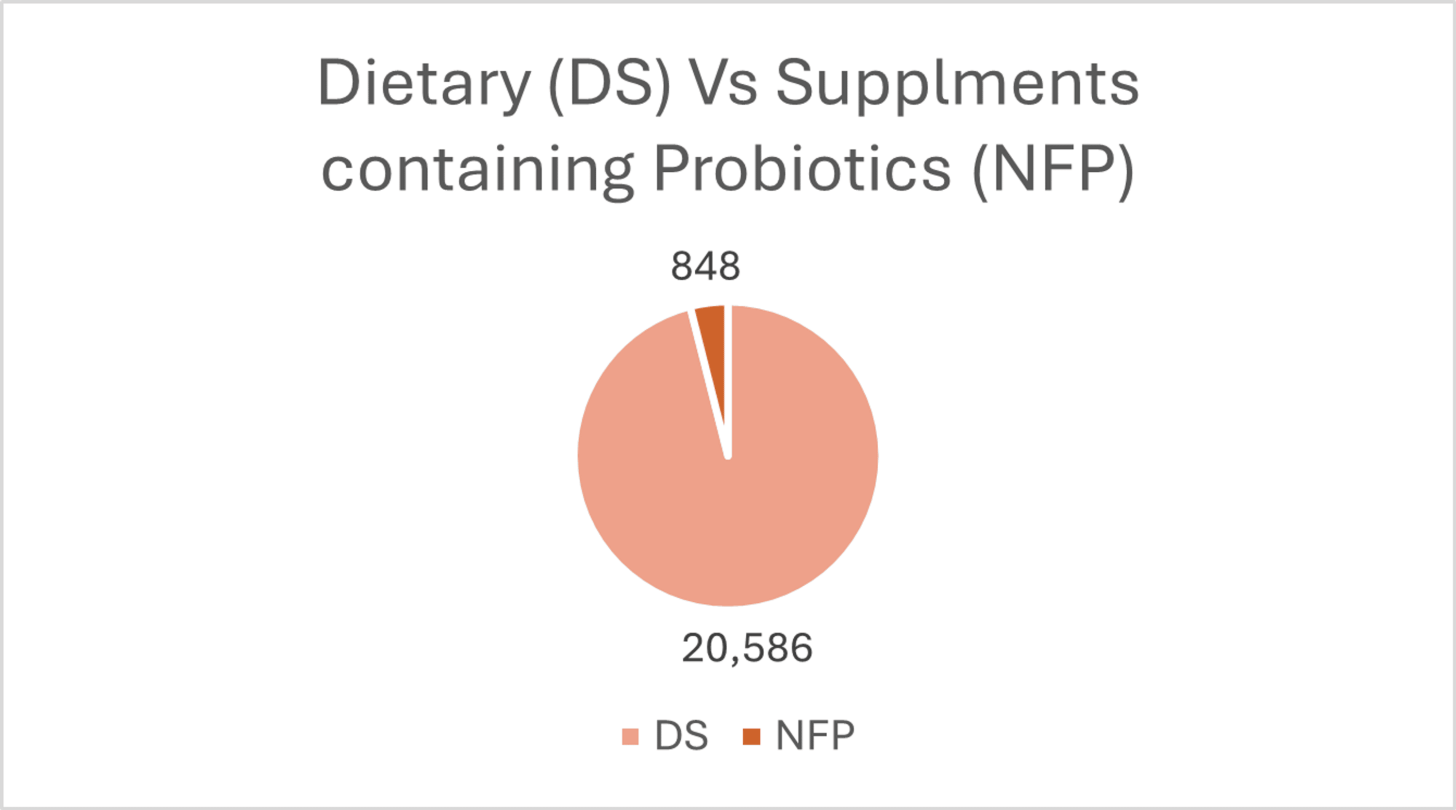
Number of participants using non-food sources of probiotics, prebiotics, or synbiotics (NFP), based on data from the National Health and Nutrition Examination Survey (1999–2018).

**Figure 6:**
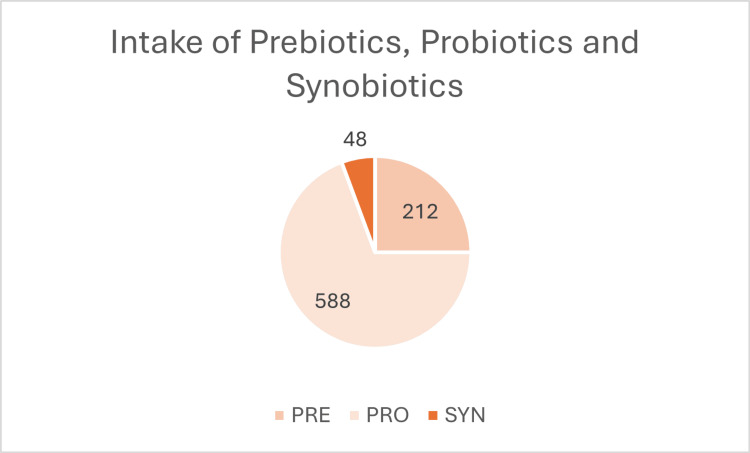
Pie chart illustrating the distribution of dietary supplement use among adults. Created the charts using Excel.

A cohort study supports this increase in the probiotic intake. It involved 53,333 adults from the National Health and Nutrition Examination Survey (1999-2018), which found that 20,586 (38.6%) used dietary supplements. Of these, only 848 (1.6%) reported using non-food prebiotics, probiotics, or synbiotics within a month: 212 used prebiotics, 588 used probiotics, and 48 used synbiotics [29]. The data is demonstrated in the following charts which presented in Figures 4-6.

Another study found that milk exosomes are promising natural nanoparticles for the delivery of probiotics. The mExo@DSPE-PEG-PBA formulation effectively encapsulates probiotics such as Akkermansia muciniphila and B. animalis subsp. lactis BB-12, and L. plantarum Q7, shielding them from gastrointestinal challenges and enhancing their adhesion. This system presents a potential new carrier for improving probiotic viability and colonization [30]. The oral administration of probiotics is a promising strategy to regulate the host intestinal flora balance and improve health [30].

Mechanisms of Probiotics in the Prevention of CAD

Cardiovascular disease is known to be the major cause of death across the globe, often as a result of underlying CAD.

The research established that dysbiosis is significantly linked to the development of CAD through mechanisms like increased gut permeability and metabolic endotoxemia, mainly by the production of LPS by the gram-negative bacteria, which can breach the intestinal barrier and then find their way to the bloodstream, serving as a crucial mediator of chronic inflammation [19].

Metabolic endotoxemia is characterized by persistently elevated plasma LPS levels, which may establish a link between dysbiosis and CAD. LPS activates toll-like receptors (TLRs), resulting in endothelial damage and heightened expression of adhesion molecules, such as CD14, on inflammatory cells while promoting the release of pro-inflammatory cytokines. Additionally, LPS may play a role in plaque formation and the advancement of atherosclerosis [19].

An RCT concluded that 12 weeks’ probiotic supplementation (L. rhamnose) on 44 patients with CAD resulted in a positive effect on endotoxemia and chronic inflammation in CAD patients including a marked reduction in IL1-beta concentration and LPS levels which improved the cardiovascular-related factors [19].

Another six-month RCT was conducted to investigate the added benefits and mechanism of the probiotic strain, Bifidobacterium lactis Probio-M8, in alleviating CAD with a conventional regimen. Sixty patients with CAD were randomly divided into a probiotic group (n = 36; received Probio-M8, atorvastatin, and metoprolol) and a placebo group (n = 24; placebo, atorvastatin, and metoprolol). Conventional treatment significantly improved the Seattle Angina Questionnaire (SAQ) scores of the placebo group after the intervention. However, the probiotic group achieved even better SAQ scores at day 180 compared with the placebo group (P < 0.0001) [16].

Mechanism of Probiotics in the Prevention of Hypercholesterolemia

Probiotics have demonstrated potential in helping manage hypercholesterolemia and associated metabolic disorders. One of the most beneficial probiotics in lipid metabolism and restoring the gut flora is Lactobacillus paracasei TISTR 2593, which was used in a 90-day RCT and has indicated potential in lowering LDL-C levels in individuals with hypercholesterolemia. The results showed a significant change in the composition of the gut microbiome and an increase in microbial abundance in the treatment individual group, demonstrating the promising therapeutic beneficial effect of this probiotic strain for managing hypercholesterolemia and lowering cardiovascular risk [14].

Another study demonstrated that probiotics are linked to a decrease in total cholesterol and triglycerides and an increase in HDL cholesterol, with no significant impact on LDL cholesterol. The cholesterol-lowering effects of probiotics may result from several mechanisms, including bile salt deconjugation, modulation of lipid metabolism, decreased intestinal cholesterol absorption through co-precipitation with bile salts, incorporation of cholesterol into probiotic membranes, conversion of cholesterol to coprostanol, and inhibition of the small intestine brush border membrane cholesterol transporter Niemann-Pick C1-like 1 (NPC1L1) in enterocytes [17].

Mechanism of Probiotics in the Prevention of Obesity

Obesity is a major chronic illness that is significantly linked to the increased risk of cardiovascular issues. A diet high in fats detrimentally influences gut microbiota, leading to a decline in beneficial bacterial populations such as Bifidobacteria, Tenericutes, Bacteroidetes, Bacteroides, Lactobacillus, Roseburia, Eubacterium rectale, and Blautia coccoides. In contrast, such a diet boosts the presence of Firmicutes, Acinetobacteria, Proteobacteria, Deltaproteobacteria, Gammaproteobacteria, and harmful pathobionts like Staphylococcus spp., Odoribacter spp., Neisseria spp., and Propionibacterium spp. This imbalance in the gut bacteria is called dysbiosis, which affects overall health, including the cardiovascular system [31].

Moreover, obese individuals with metabolic disorders tend to show reduced microbiome diversity compared to those who are merely obese. Research suggests that weight loss can increase the proportion of Bacteroidetes in the gut while adopting a high-fiber, low-fat diet, which correlates with reducing Firmicutes. Given that obesity is a persistent condition substantially raising the likelihood of cardiovascular events, dysbiosis plays a vital role in disrupting metabolic and energy balance. High-fat diets adversely affect the gut microbiota by diminishing beneficial bacteria and amplifying harmful microbial populations [31].

People who are obese and also have metabolic disorders generally exhibit a lower diversity in their gut microbiome. In contrast, weight loss has been linked to increased beneficial Bacteroidetes. Dietary strategies prioritizing high fiber and lower fat content may further reduce Firmicutes levels [31]. This relationship underscores the potential for probiotics to alter the gut microbiota, which may help prevent obesity and its related health challenges [31].

The mechanism of probiotics that can affect obesity, via modulation of the gut microbiota composition, further improves the effect of dysbiosis that is usually prominent in obese individuals; moreover, probiotics increase the production of SCFAs and decrease the LPS. SCFAs stimulate enterocyte receptors and secretion of GLP1 and peptide YY (PYY), which in turn regulate energy homeostasis. Probiotics also stimulate the release of GLP2, which improves the expression of tight junction proteins and the gut barrier function, ultimately improving the inflammatory cascade and insulin resistance in obese individuals [20].

A meta-analysis study that used probiotics supplements together with dairy products for more than 12 weeks at the dosage level of 10^7^ to 10^11^ Colony Forming Units (CFU/day) on 16676 overweight/obese adults (BMI >25) showed a bigger reduction in body weight (BW), body fat mass (BFM), and body mass index (BMI) [20].

An RCT was conducted on humans to investigate the effect of Bifidobacterium longum BB536 and Bifidobacterium breve MCC1274 on 100 participants with a BMI above 23, over a 16-week treatment period. The outcome was satisfactory and supported the effective role of probiotics in controlling the obesity risk through the reduction of the visceral fat area, total fat area, and BMI. At the same time, there was no change in the subcutaneous fat area. Furthermore, the probiotic group (with the intake of fermented milk containing B. longum BB536) showed a marked reduction in triglyceride serum levels compared to the control (with the intake of fermented milk without B. longum BB536) in the same study, which concluded that B. longum BB536 and B. breve MCC1274 showed efficacy in reducing the obesity risk in healthy and overweight individuals [21].

Mechanisms of Probiotics in the Prevention of T2DM

T2DM is a longstanding metabolic condition caused by insulin resistance and insufficient insulin levels in the blood. Research has proven that patients with type 2 diabetes often have alterations of the gut microbiota. These changes can potentially be managed by modifying gut microbiota. Additionally, studies have shown that probiotics may be beneficial in treating type 2 diabetes due to their properties that inhibit oxidative stress [32].

A recent study found that specific probiotic strains can enhance glucose transporters like glucose transporter type 4 in muscle and fat tissue, improving glucose uptake. Additionally, probiotics may affect bile acid metabolism, influencing lipid and glucose metabolism. Some can also produce insulin-like peptides that mimic insulin's effects, further aiding glucose uptake [17].

An RCT was conducted at the diabetic clinic at one of the research centers involving 130 participants with T2DM. The study demonstrated a significant reduction in HbA1c levels in the probiotic group (p = 0.004), underscoring the potential of probiotics to enhance glycemic control [33].

Another RCT, double-blinded, was done in China on adults diagnosed with T2DM aged 18 years old, 16 weeks treatment period with probiotics (dose of 0^8 CFU/mL of Lactobacillus four times daily (probiotic group) or an equivalent volume of inactivated Lactobacillus (placebo group). This study showed an apparent reduction in HBA1C and fasting C-peptide levels in both the probiotic and the placebo control group, with no statistically significant difference between the two groups [22].

Mechanisms of Probiotics in Hypertension

Hypertension is a chronic condition characterized by persistent blood pressure elevation. This occurs by the dysregulation of the renin-angiotensin system, which inhibits the synthesis of nitric oxide (NO) in endothelial cells, resulting in increased vascular resistance. Another contributing factor is the elevated production of reactive oxygen species, which damages blood vessels and raises blood pressure. Probiotics regulate and balance these metabolites, ultimately leading to lower blood pressure through specific mechanisms, which will be mentioned [34].

One of these mechanisms is the regulation of the gut microbiota composition and reducing the Firmicutes-to-Bacteroidetes ratio, which has been established in numerous clinical studies, suggesting a strong association between dysbiosis and hypertension, which is evidenced by a study showing the alteration in the gut microbiota after infusing Angiotensin II infusion and subsequent elevation in the blood pressure [35].

The mechanism of probiotics in lowering blood pressure involves inhibiting the production of proinflammatory cytokines, including tumor necrosis factor-α (TNF-α). They enhance the Firmicutes/Bacteroidetes (F/B) ratio and balance the gut microbiota composition [23].

LPS is a major component of the membrane of gram-negative bacteria. It activates the TLR4 receptor, contributing to the development of oxidative stress and endothelial dysfunction associated with hypertension. Research has revealed that p38 mitogen-activated protein kinase (p38MAPK) plays a significant role in the vascular inflammation induced by LPS, which is linked to the development of hypertension. Furthermore, probiotics have been found to downregulate the oxidative and kinase-dependent signaling pathways associated with LPS and TLR4, thereby producing an antihypertensive effect [36].

Fourteen meta-analyses of RCTs from 15,494 participants with hypertension revealed that probiotic supplementation effectively manages hypertension (HTN) by reducing systolic and diastolic blood pressure. Subgroup analyses indicated that factors such as mean age (over 50 years), the study population (individuals with HTN or T2DM), 10^10 CFU (colony-forming unit) or higher dosages, and intervention durations of 10 weeks or less may play significant roles in controlling the blood pressure [23].

**Figure 7:**
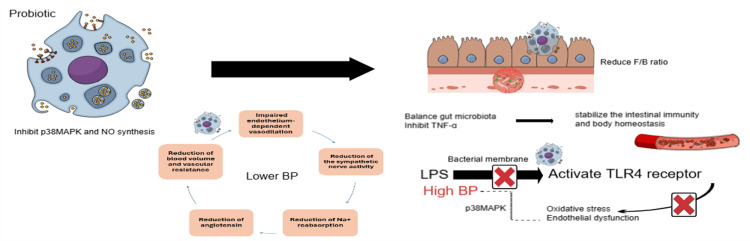
Mechanism of probiotics in lowering blood pressure by modulating the gut microbiota, stabilizing intestinal immunity by reducing the Firmicutes/Bacteroidetes (F/B) ratio, and inhibiting lipopolysaccharide (LPS). Then, it deactivates Toll-like receptor 4 (TLR4), downregulates p38 mitogen-activated protein kinase (p38MAPK), and inhibits tumor necrosis factor-α (TNF-α) along with nitric oxide (NO). The figure is created by using https://mindthegraph.com and The design of the figure incorporates features from Microsoft Windows SmartArt.

The mechanism behind the probiotic reduction of blood pressure can be established by modulating the gut microbiota composition and producing proinflammatory cytokines such as TNF-α, as the inflammation can alter the synthesis and degradation of vasoconstrictor and vasodilator (i.e., NO ), which lead to imbalance then reduce the sympathetic activity, sodium reabsorption and angiotensin then reduction of the vascular resistant and blood volume, eventually lower the blood pressure. The gut microbiota can influence blood pressure through different mechanisms, such as some bacterial genera, including Lactobacillus and Bifidobacterium, which can produce neurotransmitters, SCFA, or bacterial wall components like LPS. Probiotics also improve the F/B ratio [23]. Figure 7 illustrates the effect of probiotics on the gut microbiota, which lowers blood pressure.

The limitations of the clinical trials and studies that were discussed in the research were that many studies have been conducted on a small number of participants, which interferes with the generalizability and precision of the results. Other studies were conducted on specific populations and ethnicities, which may have variable effects on the intervention’s outcome. Considerable variations in the probiotic strains, dosages, and treatment durations make it challenging to have consistent conclusions. Moreover, some studies did not include specific individual gut microbiota samples to show if the non-responders had a specific microbiome that might impair the response. Furthermore, some trials had short-term follow-up, which interfered with evaluating the long-term effect. Few studies had limited human trials on specific metabolites. Further meticulous trials to address these limitations in future research are crucial for improving our understanding of probiotics in preventing CAD and developing advanced, scientifically supported strategies.

## Conclusions

The gut microbiota is essential in preventing CAD by protecting gut health from dysbiosis, which is strongly linked to the development of CAD. Probiotics have shown effective mechanisms to control risk factors and lower CAD. Their effect is produced through many mechanisms, such as their anti-inflammatory and antioxidative role. Other mechanisms were addressed mainly through the reduction or prevention of CAD risk factors, which are achieved by the anti-glycemic and antihypertensive effects of the probiotics and by reducing metabolic disorders. These further prevent obesity and hypercholesterolemia, which in turn signify the effective role of probiotics in preventing CAD.

Probiotics as adjunctive therapy are promising in preventing CAD in healthy individuals and reducing its progression in patients with already existing CAD. However, most studies conclude that further meticulous trials are essential to clarify their efficacy in preventing various conditions, including CAD.
